# Biomechanical comparison of different fixation methods in tibiotalocalcaneal arthrodesis: a cadaver study

**DOI:** 10.1186/s13018-023-04444-7

**Published:** 2023-12-17

**Authors:** Matthias Trost, Suzan Yarkin, Matthias Knieps, Sönke Frey, Wolfram Friedrich Neiss, Peer Eysel, Sascha Gick, Jens Dargel

**Affiliations:** 1 Department of Orthopaedics and Traumatology, Saint Josef Hospital, Ruhr University Bochum, Gudrunstrasse 56, 44791 Bochum, Germany; 2 Department of Orthopaedics and Traumatology, Protestant Hospital Cologne Weyertal, Cologne, Germany; 3 Department of Orthopaedics and Traumatology, Cologne University Hospital, Cologne, Germany; 4 Department of Orthopaedics and Traumatology, Florence Nightingale Hospital, Düsseldorf, Germany; 5 Department of Anatomy I, University of Cologne, Cologne, Germany; 6 Cologne Center for Musculoskeletal Biomechanics (CCMB), University of Cologne, Cologne, Germany; 7 Department of Traumatology, Orthopaedics and Hand Surgery, Saint Vinzenz Hospital, Cologne, Germany; 8 Department of Orthopaedics, Saint Josefs Hospital, Wiesbaden, Germany

**Keywords:** Ankle, Tibiotalocalcaneal, Arthrodesis, Nail, Plate, Screws, Biomechanical, Cadaver

## Abstract

**Background:**

Various fixation methods are available for tibiotalocalcaneal arthrodesis: nail, plate, or screws. An intramedullary bone stabilization system within a balloon catheter has not previously been used in tibiotalocalcaneal arthrodesis. The aim of this study was to compare the stability of these techniques.

**Methods:**

Twenty-four lower legs from fresh-frozen human cadavers were used. Tibiotalocalcaneal arthrodesis was performed with a retrograde nail, a lateral locking plate, three cancellous screws, or an intramedullary bone stabilization system. The ankles were loaded cyclically in plantarflexion and dorsiflexion.

**Results:**

For cyclic loading at 125 N, the mean range of motion was 1.7 mm for nail, 2.2 mm for plate, 6.0 mm for screws, and 9.0 mm for the bone stabilization system (*P* < .01). For cyclic loading at 250 N, the mean range of motion was 4.4 mm for nail, 7.5 mm for plate, 12.1 mm for screws, and 14.6 mm for the bone stabilization system (*P* < .01). The mean cycle of failure was 4191 for nail, 3553 for plate, 3725 for screws, and 2132 for the bone stabilization system (*P* = .10).

**Conclusions:**

The stability of the tibiotalocalcaneal arthrodesis differs depending on the fixation method, with nail or plate showing the greatest stability and the bone stabilization system the least. When three screws are used for tibiotalocalcaneal arthrodesis, the stability is intermediate. As the biomechanical stability of the bone stabilization system is low, it cannot be recommended for tibiotalocalcaneal arthrodesis.

## Background

Tibiotalocalcaneal arthrodesis is used for operative treatment of severe degenerative and traumatic pathologies of the ankle and hindfoot. Implants commonly used for tibiotalocalcaneal arthrodesis include nail, plate, and screws [1–4].

An intramedullary bone stabilization system based on a light-curable monomer inside a balloon catheter was developed specifically for osteoporotic bone. Initial studies investigating the intramedullary bone stabilization system are available, but it has not yet been used for tibiotalocalcaneal arthrodesis [5–8].

Previous biomechanical studies have compared a retrograde nail with different plates (lateral blade plate, lateral locking plate, lateral locking plate with an augmentation screw, posterolateral locking plate) for tibiotalocalcaneal arthrodesis in fresh-frozen cadavers [9–13]. Some of these studies reported greater stability for a plate, while other studies did not observe any difference in stability between nail and plate for tibiotalocalcaneal arthrodesis [9–13]. A few published biomechanical studies have compared a retrograde nail with different screw constructs for tibiotalocalcaneal arthrodesis [14–16]. In fresh-frozen cadavers, Berend et al. observed significantly greater stiffness in the tibiotalocalcaneal arthrodesis with a retrograde nail in comparison with two cancellous screws [14]. In Sawbone studies, similar stability in the tibiotalocalcaneal arthrodesis has been reported with an uncompressed nail and a three-screw construct [15, 16].

The aim of the present biomechanical study was to determine whether there are any differences in stability between a retrograde nail, a lateral locking plate, three cancellous screws, or an intramedullary bone stabilization system for tibiotalocalcaneal arthrodesis. It was hypothesized that there would be no differences in stability between these techniques. The primary aim was to compare the range of motion during cyclic loading. A secondary aim was to compare the cycles of failure.

## Mehods

The study was conducted with the approval of the local ethics committee (ref. no. 15–364). Fourteen matched pairs of fresh-frozen human cadaver lower legs from consenting informed donors were used for the study (Table 1). The bone mineral density in each specimen was measured in the posterior third of the calcaneus using dual X-ray absorptiometry (DEXA; Lunar iDXA, GE Healthcare, Chicago, USA) [17, 18]. Radiographs of the specimens in anteroposterior and lateral projections were taken in order to exclude any osseous pathology. Four of the 28 specimens had to be excluded owing to osseous pathologies (Table 1).

**Table 1:** Study population

Specimen no	Age (years)	Sex	Right lower leg	Left lower leg
1	68	Male	Nail	Excluded
2	73	Male	Excluded	Bone stabilization system
3	89	Male	Nail	Bone stabilization system
4	77	Female	Nail	Bone stabilization system
5	61	Male	Bone stabilization system	Nail
6	73	Female	Bone stabilization system	Nail
7	81	Male	Bone stabilization system	Nail
8	85	Female	Plate	Screws
9	87	Female	Plate	Excluded
10	86	Female	Excluded	Screws
11	80	Female	Plate	Screws
12	95	Female	Screws	Plate
13	66	Female	Screws	Plate
14	82	Male	Screws	Plate

The tibia and fibula were shortened to a length of 30 cm measured from the ankle joint [19, 20]. Disarticulation of the Chopart joint was performed [19, 20]. Soft tissues were removed except for the membrana interossea, the syndesmosis, and the ankle ligaments [19, 20]. The joint surfaces were left intact [19, 20]. The specimens were wrapped in saline-soaked compresses and stored at − 20 °C between experiments. For the experiments, the specimens were thawed at room temperature for 12 h.

**Fig. 1:**
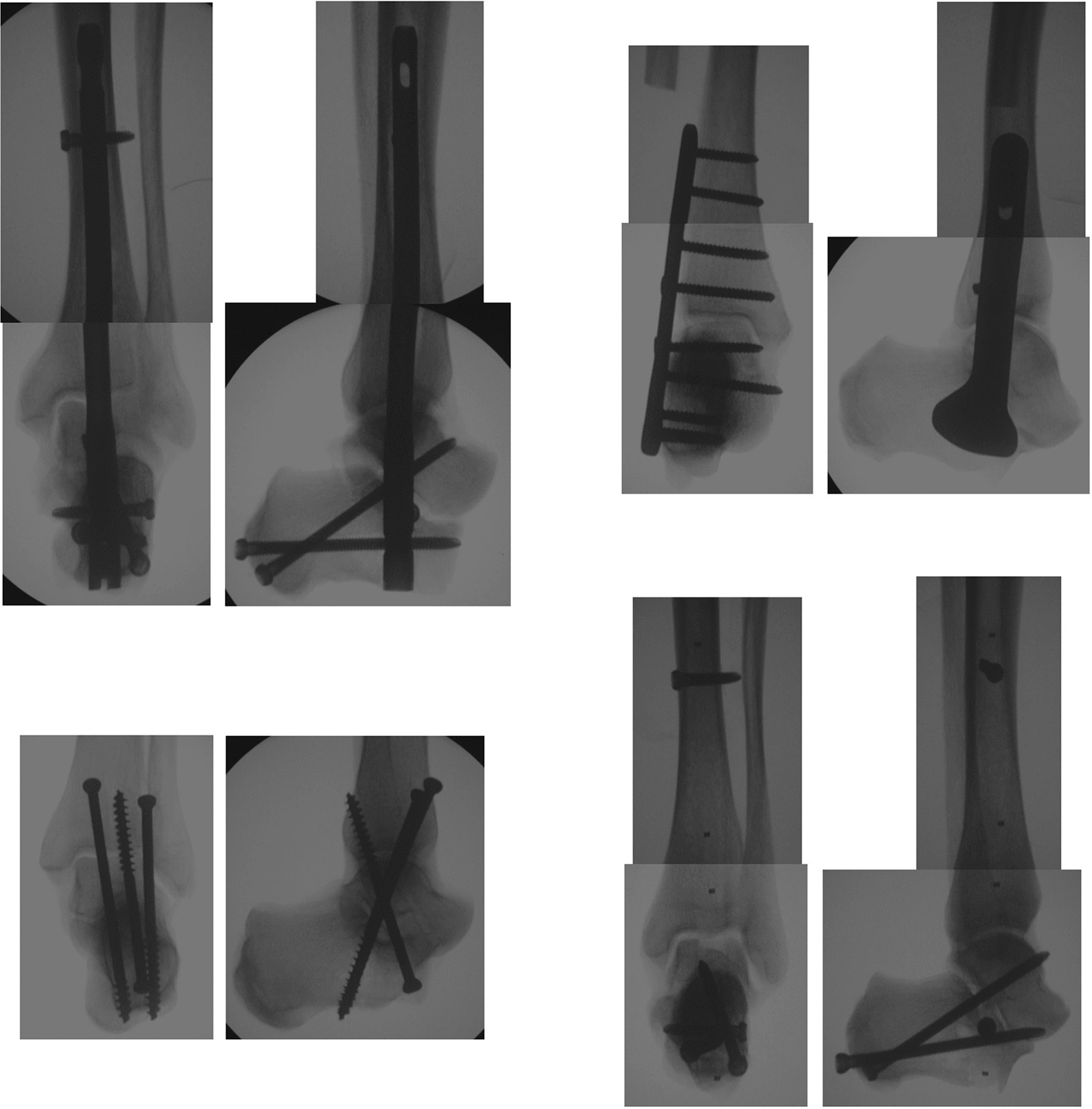
Radiographs in anteroposterior and lateral projections after tibiotalocalcaneal arthrodesis with nail, plate, screws, or the bone stabilization system

The 24 specimens were assigned equally to one of four groups (n = 6 for each group): Nail, plate, screws, or bone stabilization system (Table 1). Tibiotalocalcaneal arthrodesis was carried out by the second author. In the nail group, the medullary cavity of the tibia was reamed up to a diameter of 11 mm via the calcaneus and talus. An intramedullary nail (Trigen Hindfoot Fusion Nail, 10 × 200 mm, Smith and Nephew, Memphis, Tennessee, USA) was inserted retrogradely and locked statically via the drill guide using four 5.0-mm locking screws. In the plate group, the distal 10 cm of the fibula was resected. A locking plate (Peri-Loc Ankle Fusion Large Fragment System, length 120 mm, Smith & Nephew, Memphis, Tennessee, USA) was positioned laterally and fixed with one 4.5-mm cortical screw and eight 4.5-mm locking screws in neutral mode. In the screws group, three 6.5-mm partially threaded cancellous screws (DePuy Synthes, Zuchwil, Switzerland; steel) were used for tibiotalocalcaneal arthrodesis. In accordance with the method described by Mückley et al. in Sawbones, two screws were inserted in parallel from the ventral tibia to the dorsal calcaneus, and the third screw in between from the ventral calcaneus to the dorsal tibia [16]. In the bone stabilization system group, the medullary cavity of the tibia was reamed up to a diameter of 11 mm via the calcaneus and talus, as in the nail group. Using the drill guide from the nail group and a 4.0-mm drill bit, four holes were drilled at the same positions as in the nail group. After cleaning of the medullary cavity with a brush, the bone stabilization system (IlluminOss Medical, East Providence, Rhode Island, USA; size 13 × 180 mm; www.illuminoss.com) was positioned in the medullary cavity. The photodynamic liquid monomer was infused into the balloon and cured using the light system for 600 s. After drilling of the previously created four holes through the cured monomer, the bone stabilization system was locked with four 5.0-mm locking screws, as also used in the nail group. In all of the groups, radiographs were taken in anteroposterior and lateral projections to verify correct implant positioning (Fig. 1).

**Fig. 2:**
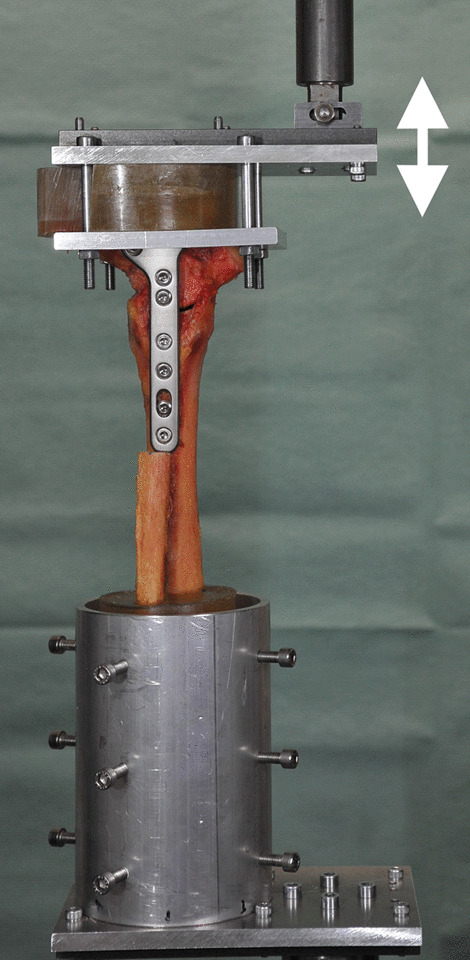
The experimental set-up for mechanical testing

The experimental set-up for mechanical testing is based on the method described by Richter et al. and Evers et al. [19, 20]. The base of the calcaneus and the proximal 15 cm of the remaining tibia and fibula were embedded in cold curing resin (Technovit® 4004, Heraeus Kulzer GmbH, Wehrheim, Germany) [19–21]. Each specimen was tested in plantarflexion and dorsiflexion, with load application via a lever of 8 cm in length with a materials testing machine (Z010, Zwick Roell, Ulm, Germany) (Fig. 2) [19, 21, 22]. The preload was 5 N. The specimens were loaded at a displacement rate of 10 mm/s for 1500 cycles from − 125 N to 125 N and subsequently for 3500 cycles from − 250 N to 250 N [19, 20]. Every 250 cycles, radiographs were taken in anteroposterior and lateral projections to verify correct implant positioning [19, 20]. Failure was defined as displacement (plantarflexion or dorsiflexion) of 10 mm [19]. The mode of failure was noted.

Force displacement curves were analyzed in Microsoft Excel 2007 (Microsoft Corporation, Redmond, Washington, USA). Statistical analysis was carried out using IBM SPSS Statistics for Windows, version 22.0 (IBM Corporation, Armonk, New York, USA). The Shapiro–Wilk test was used to check the normal distribution of data. The results for the four groups were compared using analysis of variance (ANOVA) for normally distributed data and the Kruskal–Wallis test for nonnormally distributed data. The correlation between bone mineral density and cycle of failure was evaluated using Pearson correlation. Statistical significance was set at the 5% level (*P* ≤ 0.05).

## Results

Table 2 shows the results relative to bone mineral density and mechanical testing. The mean bone mineral density did not differ significantly between the four groups, at 0.64 g/cm^2^ (range 0.49–0.77 g/cm^2^) in the nail group, 0.56 g/cm^2^ (range 0.38–0.78 g/cm^2^) in the plate group, 0.58 g/cm^2^ (range 0.41–0.78 g/cm^2^) in the screws group, and 0.64 g/cm^2^ (range 0.49–0.83 g/cm^2^) in the bone stabilization system group (*P* = 0.61).

**Table 2:** Results (mean and range) relative to bone mineral density and mechanical testing

	Nail	Plate	Screws	Bone stabilization system	*P*
n	6	6	6	6	
Bone mineral density (g/cm^2^)	0.64 (0.49–0.77)	0.56 (0.38–0.78)	0.58 (0.41–0.78)	0.64 (0.49–0.83)	0.61
Range of motion during 125 N loading (mm)	1.7 (1.1–2.4)	2.2 (1.2–3.3)	6.0 (3.4–14.1)	9.0 (5.2–11.5)	< 0.01*
Range of motion during 250 N loading (mm)	4.4 (2.0–6.1)	7.5 (2.8–12.0)	12.1 (8.3–15.8)	14.6** (13.1–16.0)	< 0.01***
Number of failed specimens during loading at 125 N	1	0	1	2	
Number of failed specimens during loading at 250 N	0	3	1	4	
Number of specimens without failure	5	3	4	0	
Cycle of failure	4191 (146–5000)	3553 (1674–5000)	3725 (501–5000)	2132 (520–4501)	0.10

*P < .01 for: nail versus bone stabilization system, plate versus bone stabilization system

**The bone stabilization system group was not included in the statistical analysis. Due to prior failure, there were results from only two specimens for 250 N loading

***P < .01 for: nail versus screws

**Fig. 3:**
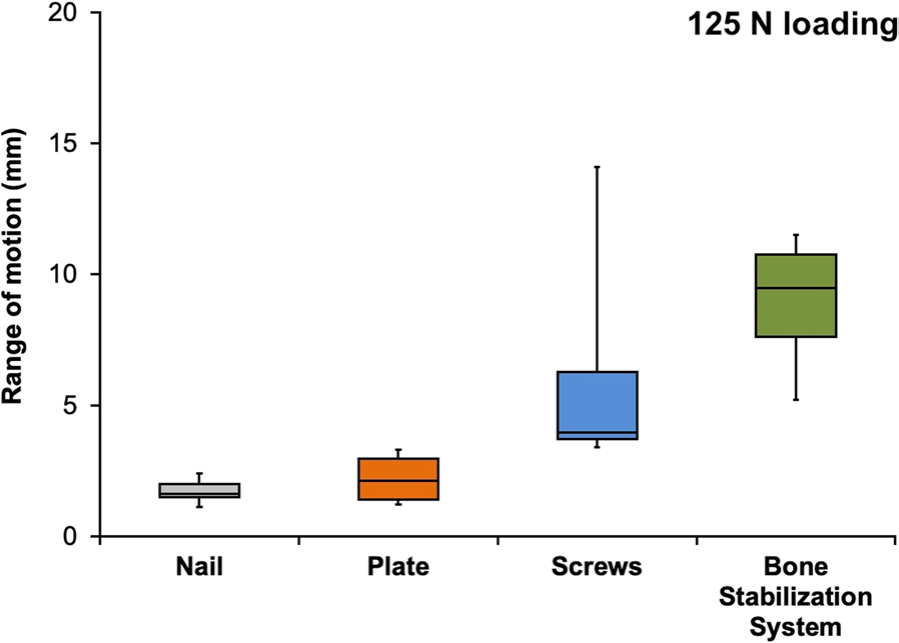
Box plot for the range of motion during 125 N loading, showing medians and quartiles (nail group n = 5; other groups n = 6 per group)

The mean range of motion during 125 N loading differed significantly between the four groups, at 1.7 mm (range 1.1–2.4 mm) for nail, 2.2 mm (range 1.2–3.3 mm) for plate, 6.0 mm (range 3.4–14.1 mm) for screws, and 9.0 mm (range 5.2–11.5 mm) for the bone stabilization system (*P* < 0.01). There was a significantly higher range of motion during 125 N loading in the bone stabilization system group in comparison with the nail group and the plate group (*P* < 0.01). Figure 3 shows the medians and quartiles for the range of motion during 125 N loading in the four groups.

**Fig. 4:**
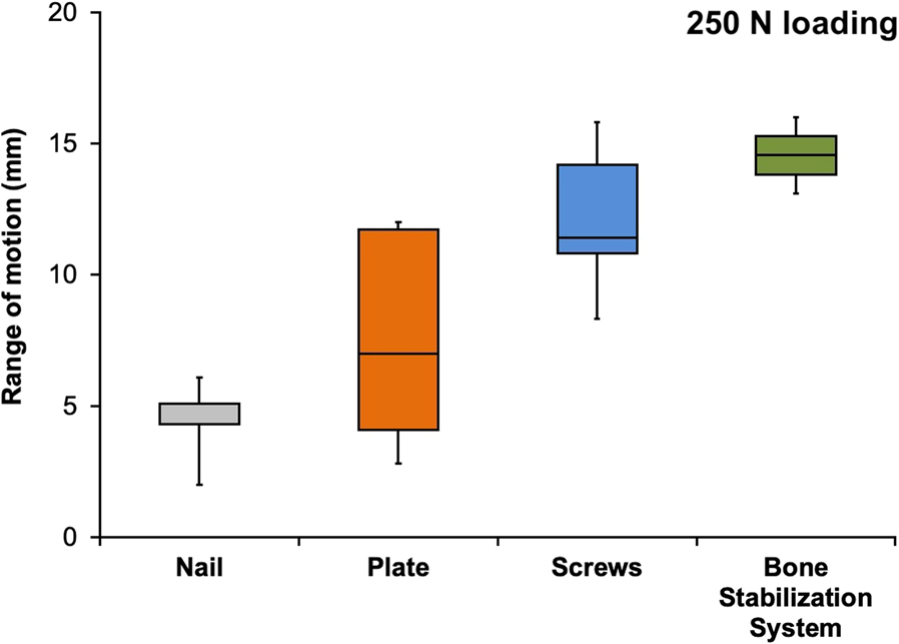
Box plot for the range of motion during 250 N loading, showing medians and quartiles (bone stabilization system group n = 2; other groups n = 5 per group)

The mean range of motion during 250 N loading differed significantly between three of the groups, at 4.4 mm (range 2.0–6.1 mm) for nail, 7.5 mm (range 2.8–12.0 mm) for plate, and 12.1 mm (range 8.3–15.8 mm) for screws (*P* < 0.01). There was a significantly higher range of motion during 250 N loading in the screws group in comparison with the nail group (*P* < 0.01). In the bone stabilization system group, the mean range of motion during 250 N loading was 14.6 mm (range 13.1–16.0 mm). Due to prior failure, it could only be calculated for two specimens in the bone stabilization system group. The bone stabilization system group was therefore not included in the statistical analysis of 250 N loading. Figure 4 shows the medians and quartiles for the range of motion during 250 N loading in the four groups.

The mean cycle of failure did not differ significantly between the four groups, at 4191 (range 146–5000) for nail, 3553 (range 1674–5000) for plate, 3725 (range 501–5000) for screws, and 2132 (range 520–4501) for the bone stabilization system (*P* = 0.10). Specimens failed as a result of implant loosening. In the bone stabilization system group, the screws broke out of the cured monomer. Table 3 shows the correlation between bone mineral density and cycle of failure. The Pearson correlation coefficient was 0.55 for all groups (*P* < 0.01) and 0.85 in the screws group (*P* = 0.03). The other groups did not show any significant correlation between bone mineral density and cycle of failure.

**Table 3:** Correlation between bone mineral density and cycle of failure

	n	Pearson correlation coefficient	*P*
Nail	6	0.73	0.10
Plate	6	0.59	0.22
Screws	6	0.85	0.03
Bone stabilization system	6	0.49	0.32
All groups	24	0.55	< 0.01

## Discussion

This cadaver study compared the biomechanical properties after tibiotalocalcaneal arthrodesis with a retrograde nail, a lateral locking plate, three cancellous screws, and an intramedullary bone stabilization system based on a light-curable monomer inside a balloon catheter.

The present results show that a high degree of stiffness in the tibiotalocalcaneal arthrodesis can be achieved with a retrograde nail or a lateral locking plate. Ohlson et al. did not observe any differences in the rigidity of the tibiotalocalcaneal arthrodesis in fresh-frozen cadavers in comparison with a locking plate with a nail [12]. The locking plate investigated by Ohlson et al. used more screws for fixation in the talus and the calcaneus compared with the locking plate investigated in the present study [12]. In fresh-frozen cadavers, O’Neill et al. noted greater rigidity in the tibiotalocalcaneal arthrodesis when using a locking plate with an additional augmentation screw in comparison with a nail [11]. This result can be explained by the additional augmentation screw from the talus to the tibia used by O’Neill et al. [11]. In fresh-frozen cadavers, Gutteck et al. studied a posterolateral plate for tibiotalocalcaneal arthrodesis and detected greater stiffness in comparison with a nail [13]. The reason for this result might be the posterolateral position of the plate investigated by Gutteck et al. [13]. In the present study, a low level of stiffness in the tibiotalocalcaneal arthrodesis was observed with the bone stabilization system. This was the first use of the bone stabilization system for tibiotalocalcaneal arthrodesis. Biomechanical data for comparison are therefore not available. Nevertheless, the characteristics of the intramedullary bone stabilization system, which include infusion of a photodynamic liquid monomer into a balloon and curing by light, probably did not achieve the stability needed for tibiotalocalcaneal arthrodesis. The stiffness of a tibiotalocalcaneal arthrodesis with three screws was intermediate. For 250 N loading, it was significantly lower in comparison with the nail. Similarly, Berend et al. reported significantly less stiffness for two cancellous screws in comparison with a nail for tibiotalocalcaneal arthrodesis in fresh-frozen cadavers [14]. Based on the present results and the study by Berend et al., it can be concluded that cancellous screws provide lower stability in tibiotalocalcaneal arthrodesis compared with a nail [14].

The mean cycle of failure for a tibiotalocalcaneal arthrodesis with the bone stabilization system was 2132. This result was lower in comparison with a nail, with a mean cycle of failure of 4191, but the difference was not significant. After tibiotalocalcaneal arthrodesis with a nail, one of six specimens failed during cyclic loading. In contrast, all six specimens failed after tibiotalocalcaneal arthrodesis with the bone stabilization system. However, it should be borne in mind here that the specimen after tibiotalocalcaneal arthrodesis with a nail failed at cycle no. 146 out of a total of 5000 possible cycles. This was the first specimen in all four of the groups that failed during cyclic loading. One possible explanation for this might be the bone mineral density of 0.49 g/cm^2^, the lowest of all specimens in the nail group.

Biomechanical properties are influenced by bone mineral density, which was therefore measured in every specimen used in the present study. The aim with specimen distribution was to achieve a similar mean bone mineral density in the groups, and this was accomplished in this study. There was a significant correlation between bone mineral density and cycle of failure in all groups and in the screws group. A similar result was reported by Klos et al. for tibiotalocalcaneal arthrodesis with a nail in fresh-frozen cadavers [21]. Gutteck et al. observed a linear relationship between stiffness and bone mineral density for tibiotalocalcaneal arthrodesis with a posterolateral plate in fresh-frozen cadavers [13].

An important question is what level of stability is needed in tibiotalocalcaneal arthrodesis to achieve bone healing. There is no exact answer for this question in the literature. Nevertheless, it is assumed that the bone stabilization system cannot achieve bone healing in tibiotalocalcaneal arthrodesis because of its low stability.

The present study has several limitations. An important one is the relatively small number of specimens investigated. This is a problem with most biomechanical studies, as there are only limited numbers of fresh-frozen cadaver specimens available. This biomechanical study investigated the primary stiffness after tibiotalocalcaneal arthrodesis. The effects of bone healing cannot be determined in a biomechanical cadaver model. The results of the study are therefore only applicable to the day of operation.

## Conclusions

In conclusion, the stability of the tibiotalocalcaneal arthrodesis differs depending on the fixation method, with nail or plate showing the greatest stability and the bone stabilization system the least. When three screws are used for tibiotalocalcaneal arthrodesis, the stability is intermediate. As the biomechanical stability of the bone stabilization system is low, it cannot be recommended for tibiotalocalcaneal arthrodesis. There was a significant correlation between bone mineral density and cycle of failure in all groups and in the screws group.

## Data Availability

The datasets used and analysed during the current study are available from the corresponding author on reasonable request.
